# Using dialogues to increase positive attitudes towards COVID-19 vaccines in a vaccine-hesitant UK population

**DOI:** 10.1098/rsos.220366

**Published:** 2022-10-12

**Authors:** Charlotte O. Brand, Tom Stafford

**Affiliations:** Department of Psychology, University of Sheffield, Sheffield S1 1HD, UK

**Keywords:** chatbot, COVID-19 vaccines, dialogue, attitude change, counterarguments

## Abstract

Recently, Altay *et al.* (Altay *et al*. 2021. *J. Exp.Psychol.: Appl.* (doi:10.1037/xap0000400)) showed that 5 min of interaction with a chatbot led to increases in positive COVID-19 vaccination attitudes and intentions in a French population. Here we replicate this effect in a vaccine-hesitant, UK-based population. We attempt to isolate what made the chatbot condition effective by controlling the amount of information provided, the trustworthiness of the information and the level of interactivity. Like Altay *et al.*, our experiment allowed participants to navigate a branching dialogue by choosing questions of interest about COVID-19 vaccines. Our control condition used the same questions and answers but removed participant choice by presenting the dialogues at random. Importantly, we also targeted those who were either against or neutral towards COVID-19 vaccinations to begin with, screening-out those with already positive attitudes. Replicating Altay *et al.*, we found a similar size increase in positive attitudes towards vaccination, and in intention to get vaccinated. Unlike Altay *et al.*, we found no difference between our two conditions: choosing the questions did not increase vaccine attitudes or intentions any more than our control condition. These results suggest that the attitudes of the vaccine hesitant are modifiable with exposure to in-depth, trustworthy and engaging dialogues.

## 1. Introduction

Communicating the effectiveness, safety and necessity of vaccination is arguably one of science communication's most important and emblematic challenges. Appropriately, huge amounts of attention and research effort have been directed towards how to increase COVID-19 vaccination uptake. Owing to the urgency and impact of the problem, a multi-pronged attack is warranted, and thus research rightly spans many different strategies, from pre-empting misinformation on social media [1], presenting information on the comparison of COVID-19 symptoms to vaccination side-effects [2], presenting information on the timeline of vaccine development [2], different styles of myth-busting [3], the use of social norms [4], framing messaging in terms of individual risk preferences [5], and even chatbots [6], all with varying levels of success.

Although chatbots are usually used for aiding the completion of tasks, for example navigating website frequently asked questions (FAQs) or purchasing personalized items (train tickets and flights), interest is growing in their ability to create engaging, human-like dialogue. One way in which chatbots could be used for attitude change is their ability to deliver counterarguments to common questions or concerns. The use of chatbots to change attitudes has previously been explored in the context of genetically modified organism (GMO) attitudes [7]. The authors found, that the chatbot increased positive attitudes towards GMO foods compared to two comparisons: (i) a short description of GMOs, and (ii) a description of the consensus scientific view, but, it did not have a positive effect compared to a third condition: a counterargument condition. In this counterargument condition, participants were exposed to all GMO beliefs and counterarguments at once, rather than choosing which counterarguments to interact with. This suggested that providing access to counterarguments, rather than the choice of information, was the driving factor behind the success of the chatbot. The authors also found that the positive attitudes were mediated by time spent in the conditions, and that people spent on average longer in the counterargument condition. Crucially, they also found that in the chatbot condition, for three out of four arguments, the best predictor for selecting a given argument was how negative their initial view towards it was, suggesting participants did seem to select arguments based on their concerns.

The idea that the choice of information is important chimes with research into people's apparent preference for choosing their own actions, making their own decisions and choosing what path to take, even foregoing monetary rewards to retain agency [8]. Domains as diverse as animal learning and robotic control have shown the importance of intrinsic motivations for agency, curiosity and control for understanding and enabling complex behaviour [9]. It is reasonable, therefore, to assume that a chatbot experience may be engaging and by turn convincing because it supports the participant in playing an active role in the dialogue, making choices about the aspects of the topic they explore.

As well as ensuring the information aligns with participant's interests, it is also crucial to communicate trust for successful public health communication [10]. Eiser *et al*. [11] studied public attitudes in response to communication about pollution where they lived, and found that those who didn't trust scientific communication tended to doubt that the scientists had their own interests at heart, rather than doubt their expertise. Furthermore, high trust in information from other sources, such as family and friends, was not based on a misperception of greater expertise, but on the (arguably accurate) perception that these groups had their interests at heart. Indeed, low trust in government is consistently one of the strongest predictors of vaccine hesitancy [12]. Evidently the effectiveness of communication interventions to increase vaccination intentions may be affected by how trustworthy the intervention is deemed to be.

This paper replicates recent success in increasing positive attitudes towards, and intentions to take, COVID-19 vaccines by using a chatbot [6]. The chatbot study included participants from a random sample of French adults, whereas here we recruit vaccine-hesitant, UK-based adults only, and attempt to dissect what in particular it was about the chatbot that was effective. In particular, we wanted to test if the choice of information is a crucial factor driving the effectiveness of the chatbot. The French chatbot enabled participants to select frequently asked questions about COVID-19 vaccinations and then presented participants with answers to those questions. The chatbot would then present follow-up questions and further counterarguments. This was compared to a control condition in which the participants read 90 words of standard information from a government website. We wanted to investigate a variety of factors that may have been responsible for the increase in vaccination attitudes and intentions, such as (i) the amount of information, (ii) the time spent with the information, (iii) the interactivity or choice of information, and (iv) the trustworthiness of the information. The ‘chatbot’ condition manifestly allowed participants greater choice, but it also exposed participants to a greater amount of information, and they tended to spend more time engaged as a consequence. The chatbot condition also included content on the trustworthiness of the information being presented, whereas the control condition did not. As such, it is not clear which underlying factors drive the observed effect.

To address our question of what drove the increase in positive vaccination attitudes and intentions, the current study uses the same information as Altay *et al*. [6] but deploys two conditions in which the only difference is the interactivity of the information, i.e. the ability to choose which information to view. This allows us to directly test whether the interactivity of the information was a driving factor behind the success of the chatbot, by comparing the results of our control and choice conditions. The amount of information (number of words), time spent on the information and indicators of the trustworthiness of the information are the same in both our control and our choice conditions, allowing us to indirectly test whether these affect the success of the intervention, by comparing our results to Altay *et al*.'s [6] results.

## 2. Method

### 2.1. Pre-registered hypotheses

Our hypotheses, predictions and analyses were pre-registered before data collection at https://osf.io/t4gav. All of our data, code and analysis scripts are available at https://github.com/lottybrand/clickbot_analysis.

We hypothesized that the choice condition would show a greater increase in positive attitudes towards COVID-19 vaccines, owing to the ability of participants to choose the information most interesting or important to them. A difference between conditions would be strong evidence that one of the important aspects of chatbots in changing attitudes is that they allow the participant to choose what information to engage with, aside from the trustworthiness and amount of information presented. This logic led to the following three pre-registered predictions:

(i) increase in willingness to have a vaccine will be predicted by condition (those in the choice condition will be more likely to show an increase in their intention to take the vaccine);
(ii) there will be an interaction between condition and time of ratings, in that vaccine attitudes will be most positive in the choice condition in the post-experiment ratings compared to the pre-experiment ratings; and
(iii) the choice condition will be rated as more engaging than the control condition.

### 2.2. Participants

Based on [6], we recruited 716 adult participants from the UK. Using the recruitment platform Prolific, we were able to prescreen for UK-based participants aged between 18 and 65 who had previously answered that they were either ‘against’ the COVID-19 vaccinations, or ‘neutral’ towards the COVID-19 vaccinations (as opposed to ‘for’ COVID-19 vaccinations). As there were 657 participants registered to Prolific who answered ‘against’ at the time of recruitment, we attempted to recruit as many from this pool as possible. We only recruited participants who answered ‘against’ for the first seven days of data collection, as per our pre-registration. This led to 479 participants who answered ‘against’ in total, and a remaining 237 who answered ‘neutral’. The mean age was 35, and 207 participants were male (502 female, two non-binary, two other, three prefer-not-to-say). Ten pilot participants were recruited on 26 April 2021 and their data used for pre-registering our analysis script only (they do not contribute data to the analyses presented here). The remaining participants were recruited between 14 and 24 May 2021.

### 2.3. Materials

The baseline questionnaire was almost identical to Altay *et al*. except that we opted to use a 7-point Likert scale as opposed to five points [13]. We asked participants to rate how strongly they agree with the following statements (from 1 = strongly disagree to 7 = strongly agree): *I think COVID-19 vaccines are safe, I think COVID-19 vaccines are effective, I think we've had enough time to develop COVID-19 vaccines, I think we can trust those who produce COVID-19 vaccines, I think it is important to be vaccinated against COVID-19*. We also asked participants if they had yet taken a dose of any COVID-19 vaccine (yes, no) and whether they would consider taking any future dose of an approved COVID-19 vaccine offered to them (yes, no, undecided).

The information we used for our two conditions was taken from the Altay *et al*. study. We translated the information into English using automated translation via Google Docs, proof-read it, updated it with the most recent information at the time using official UK National Health Service and Government sources (e.g. regarding the Astra-Zeneca blood clot news), and had the information verified and fact-checked again by an independent epidemiologist.

To mimic the main features of their chatbot—interactive choice of questions and appropriate follow-up answers—we grouped the vaccine information into five main questions: (i) is the vaccine safe? (ii) is the vaccine effective? (iii) has the vaccine been rushed? (iv) can we trust who makes the vaccine? and (v) is the vaccine necessary? Within each of the five main questions were four sub-questions. Thus, there were 20 question–answer dialogues altogether, and each participant was presented with four out of those 20. We modified each sub-question to consist of a short dialogue of between 200–500 words largely avoiding repetition. Each dialogue included a short answer and two or three follow-up question–answer pairs. (These documents along with a document recording the main changes made to each section compared to the Altay paper can be found in the electronic supplementary material and on the online repository.) Thus, our participants experienced almost identical information to Altay *et al.*, in dialogue format. As with Altay *et al.*'s study, the participant experience lacked some features of full interactive chat: in both Altay *et al.* and our study, participants were not able to freely type but chose questions from a given selection, and replies were not individually or uniquely composed. However, Altay *et al.*'s study did contain bot-like features, such as a symbol that the bot was ‘typing’, and a chat-like window, which was not present in our study.

Crucially, participants in both our control and our chatbot condition were presented with the following information about the trustworthiness of the study at the start of the condition:

‘Why should I trust you? - We are two independent researchers, Lotty Brand and Tom Stafford, funded by a research council, with no links to pharmaceutical companies or other competing interests.

We are interested in learning about people's vaccine attitudes, in providing reliable information about vaccines, and learning about people's engagement with this information.

All of the information in this study has been gathered via scientific articles and reports from the past 30 years of vaccine research, as well as the most recent studies on COVID-19. The information has been checked by experts in immunology and epidemiology as of May 12th 2021.’

By contrast, Altay *et al.*'s chatbot featured trust as one of the main question options in their chatbot condition (why should I trust you?), with a response similar to our wording above. If trust drives effectiveness of vaccine interventions then this could have driven the difference between their conditions, rather than the presence/absence of a chatbot per se. We therefore removed this question and answer from the dialogue options and inserted it at the beginning of both conditions, to ensure all participants would see it regardless of condition or choice of information. This ensured the communication of trustworthiness of our information was consistent across both conditions.

Our post-experiment questionnaire consisted of the same COVID attitude questions as the pre-experiment questionnaire, as well as questions on how engaging the experience was and how clear the information was. We also asked how often participants discuss vaccination with those who disagree with them and how often they actively learn about vaccines (e.g. via reading articles, listening to podcasts). Participants were finally asked if they would recommend our study to a friend (if yes, they were given the option to share a link via Twitter or Facebook and we recorded the proportion that did), whether they would take part again in a month's time, their age, gender and education level.

We included an attention check question among both the pre-experiment questionnaire and our post-experiment questionnaire ('We would like to check that you are paying careful attention to the information in this study. Please respond to the following item with 'somewhat agree'.). We used both of these attention check answers alongside a free-response answer to check that participants were attending to the study information, i.e. we only included those that passed both attention checks and provided coherent, relevant information in the free-response text boxes (free-response text boxes were used to collect data for a different study question).

### 2.4. Procedure

Participants were randomly assigned to either the control or choice condition. Participants in both conditions provided informed consent (ethical approval provided by the University of Sheffield) before answering the pre-exposure questionnaire, interacting with the experimental material, and finally answering a post-exposure questionnaire.

In our control condition, participants viewed four randomly chosen dialogues of between 200–500 words each, one from each of the five possible domains of vaccination concern: (i) is the vaccine safe? (ii) is the vaccine effective? (iii) has the vaccine been rushed? (iv) can we trust who makes the vaccine? and (v) is the vaccine necessary?

In our choice condition, participants were able to choose four dialogues in total of between 200 and 500 words each, one from each of the five possible domains of vaccination concern, as above. Each of the five domains contained four sub-questions. Thus, participants had four choices, with one choice from each of the five main domains each time. This ensured the amount of information that the participants were exposed to was the same as in the control condition. The information is displayed identically between the two conditions, in 200–500 word chunks at a time, so the information should be equally engaging and easy to read. This was also to ensure a similar engagement time across both conditions. These controls attempt to isolate any effect of choice of information (interactivity) as a cause of difference between the conditions.

### 2.5. Analysis

Our hypotheses, predictions and analyses were pre-registered before data collection at https://osf.io/t4gav. All of our data, code and analysis scripts are available at https://github.com/lottybrand/clickbot_analysis.

All models were run using the Rethinking package in R for Bayesian models [14]. We include model parameters based on *a priori* pre-registered hypotheses. Throughout the manuscript, we report mean model coefficients with their 89% credible intervals (CIs). Model parameters were said to have an effect on the model outcome if their 89% CI did not cross zero. Eighty-nine per cent intervals are the default CI setting for the Rethinking package, as they discourage interpreting results in terms of binary null hypothesis significance testing [14]. Ninety-five per cent intervals would not alter the interpretation of our results. When relevant, we used model comparison to aid the interpretation of results. Models were said to be a better fit to the data if their widely applicable, or Wanatabe-Aike information criterion value held the most weight out of all models tested.

Priors were chosen to be weakly regularizing, in order to control for both under- and overfitting the model to the data [14]. All models were checked for convergence using convergence criteria such as Rhat values and effective sample sizes, as well as visual inspection of trace plots.

In line with our pre-registration, we analysed whether participants increased their intention to be vaccinated using a Bayesian binomial regression model with an increase (either from ‘no’ to ‘undecided’, or from ‘undecided’ to ‘yes’) coded as a 1 (did increase intention), and all other instances as 0 (did not increase intention). We also analysed whether there was a reduction in the number of participants reporting that they would not get vaccinated, by modelling ‘no’ as 1, and all other responses as 0. The second approach was included after observing an increase in the percentage of participants changing from a ‘no’ to another category that was similar to the increase that Altay *et al.* found. Our analysis strategy differed slightly from Altay *et al.*'s, thus after we failed to find the condition effect they found, we performed an equivalent analysis to theirs. Both of these approaches are reported below.

In line with our pre-registration, when modelling Likert scale vaccination attitude responses, as well as Likert scale engagement ratings, we used ordinal categorical multi-level models, with varying intercepts for who the rater was, and for Likert scale item. This allowed us to use each Likert scale item as the unit of analysis, rather than average over several items, in accordance with recommendations on how to treat Likert scale data Liddell & Kruschke [15]. It also allows us to preserve and use all of the information and variation, and account for data clustering within items and individuals [14].

## 3. Results

### 3.1. Pre-registered hypotheses

**Figure 1.**
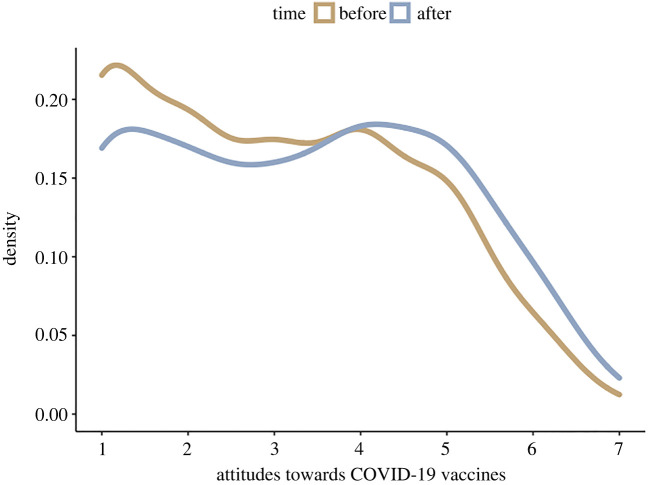
Density plot of raw vaccination attitudes before and after the experiment. 7 = strongly agree and 1 = strongly disagree with the five vaccination items: (i) vaccines are safe, (ii) vaccines are effective, (iii) they have not been rushed, (iv) those who make them can be trusted; and (v) they are necessary.

**Figure 2.**
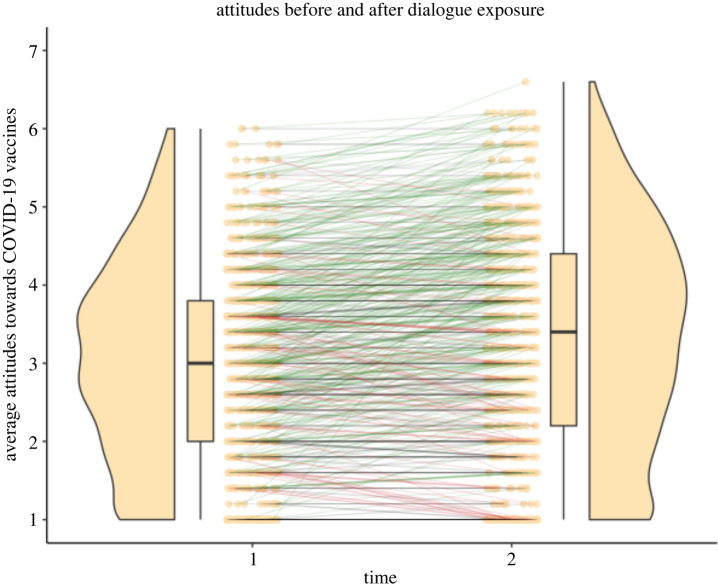
Violin plot of average vaccination attitudes before and after the experiment. 1 = negative attitudes, 7 = positive attitudes. The green lines show an increase in positive vaccination attitudes, and red lines show a decrease.

We found that participants reporting that they did not intend to get the vaccine decreased after our experiment, regardless of condition, as the number of those reporting they would not get the vaccine decreased in the post-exposure measure (mean model estimate: −0.3630144; 89% CI: −0.5304443, −0.1945209). Against prediction 1, those in the choice condition were not more likely to increase their intention to have the vaccine compared to the control condition (mean: −0.2151165; 89% CI: −0.5657521, 0.144324). These shifts in intention can be seen in table 1 and are equivalent to those found in Altay *et al.*'s chatbot condition; in Altay *et al.*'s chatbot condition, 36% of participants reported that they did not intend to get vaccinated, and this dropped to 29% afterwards. Across both our conditions, 53% reported that they did not intend to get vaccinated and this dropped to 44% afterwards (figures 1 and 2).

**Table 1.** Number of participants reporting that they do not (no), are undecided, or do (yes) intend to get vaccinated pre- and post-exposure to the dialogues in each condition.

	no	undecided	yes
choice (pre)	194	93	70
choice (post)	167	107	83
control (pre)	189	95	75
control (post)	150	124	85

We also found that vaccine attitudes increased across both conditions (mean: 1.9968003; 89% CI: 1.9261025, 2.0663825). Against prediction 2, as there was not an interaction between condition and time of ratings in our full model (mean: −0.0922423; 89% CI: −0.2379752, 0.0436521); vaccine attitudes were not most positive in the post-treatment ratings of the choice condition, but increased similarly in both conditions. This interpretation was confirmed by a model comparison approach, in which we compared models including parameters for condition, post-treatment rating, and an interaction between condition and post-treatment rating. The best-fitting model included only the experiment effect, with the worst fitting models containing the interaction effect, and just varying intercepts (null model), suggesting that the experiment effect (change across both conditions) was most informative in predicting the difference in vaccination attitudes (see the electronic supplementary material). Increase in average vaccination attitudes can be seen in the violin plot in figure 2.

This change in vaccination attitudes is displayed in figure 1, which shows the raw vaccination attitude ratings before and after the experiment. Figures displaying the differences in vaccination attitudes within different scale items (e.g. are they safe, are they effective, have they been rushed, can we trust those who makes them, are they necessary) can be found in the electronic supplementary material. These figures suggest that the majority of our sample agreed that vaccines are effective, but were undecided as to whether they are safe, and disagreed that we can trust those who produce them, that there has been enough time to produce them, and that they are necessary.

Against prediction 3, we did not find that the choice condition was rated as more engaging than the control condition (mean: 0.1581001; 89% CI: −0.0310088, 0.3483672).

### 3.2. Exploratory analysis

Overall, we found that the number of people who said that ‘no’ they would not get a vaccination when one was offered to them decreased after taking part in our experiment. Out of 571 participants reporting that they either would not, or were undecided, about getting the vaccine, 93 reported being more likely to get vaccinated after the experiment (16% increase). Out of these 93, six changed directly from a ‘no’ to a ‘yes,’ 25 went from an ‘undecided’ to a ‘yes’ and 62 went from a ‘no’ to an ‘undecided’.

As [6] found a stronger effect for those who spent the most time with the chatbot, we wanted to check whether a condition effect was present in those who spent more time with the information. The median amount of time spent viewing the information was 4 min, and we found that participants who spent above the median amount of time viewing the information (between 4 and 16 min, so between 1 and 4 min per dialogue) were more likely to increase their vaccination attitudes compared to those who spent less time viewing the information. We found a positive interaction between those who spent above the average amount of time and their post-treatment rating (mean: 0.4778941; 89% CI: 0.3416222, 0.6054146). This was confirmed by model comparison, in which the model including the interaction effect, as well as a main effect for post-treatment rating, was the best-fitting model (details in the electronic supplementary material).

When looking only at those who spent above the median amount of time with the information, we again found no effect of condition on intention to get vaccinated (mean: −0.165634; 89% CI: −0.6404874, 0.2988044).

By contrast, participants who spent above the average amount of time viewing the information were not more likely to show an increase in their intention to get vaccinated compared to the rest of the participants (mean: 0.2068541; 89% CI: −0.1461917, 0.5704015).

## 4. Discussion

We ran an experiment to test if the choice of information is a crucial factor driving the effectiveness of a COVID-19 vaccination chatbot. We recruited 716 adults based in the UK who had previously said they were ‘against’ or ‘neutral’ towards COVID-19 vaccines. Based on a chatbot experiment conducted with French participants [6], we created 20 dialogues split across the five topics: how safe the vaccines are, how effective they are, whether there has been enough time to develop them, whether we can trust who makes them, and whether they are necessary for young and healthy people. Participants were randomly assigned to two conditions; in one, they could choose the dialogues they saw (choice condition), in the other, the dialogues were randomly displayed (control condition). Overall, we found that, in both conditions, participants' vaccination attitudes and intentions shifted in a more positive direction after reading the dialogues; we found no difference between the choice and control condition. Crucially we found that participants who spent above the average (median) amount of time viewing the information (between 4 and 16 min, or between 1–4 min per dialogue) were more likely to increase their vaccination attitudes than those who spent below the average (median) amount of time viewing the information. This association between viewing time and increased change was not found for intentions.

Our results have implications in the light of recent interest in using chatbots or other interventions to increase vaccination uptake. We conclude that creating an engaging experience for participants that encourages them to spend quality time with the information is key for increasing positive attitudes towards vaccination.

The size of the shift in intentions we observed was similar to the results of the Altay *et al*. chatbot condition. In this sense, we provide a conceptual replication of their results. This is reassuring as we used identical information to theirs, only editing the information to be more appropriate for a UK-based audience and with the latest epidemiological information at the time. In both their and our experiment, we found an effect of time spent with the information, in that those who spent longer with the chatbot were more likely to increase their vaccination attitudes. This has potentially important implications for those designing public health information interventions, in that how engaging the material is (and therefore how long participants are willing to attend to the information) is crucial.

In contrast with the Altay experiment, we found no difference in effectiveness between our conditions. However, there were crucial differences between our conditions and those of Altay *et al*. The most obvious is that all of our participants saw information of the same length and quality. The fact that our conditions were equally effective then suggests that Altay *et al*.'s chatbot may have been more effective than their control condition not because there is something inherently effective about chatbots per se, but simply because it delivered more information than the control condition, as supported by Altay *et al*. [7]. The second crucial difference between our experiment and Altay *et al*.'s is that we controlled for trustworthiness of information across both of our conditions. In Altay *et al*.'s chatbot experiment, the chatbot included a question ‘why should I trust you?’ in which, if participants chose it, they saw information about who the researchers were and what their motives were. Previous research suggests trust plays a huge role in how effective science communication is [16]. The information in Altay's control condition was therefore implicitly less trustworthy than the chatbot information, given the control condition had no source and was anonymous. By contrast, both of our conditions included the ‘Why should I trust you?’ information at the start of the experiment, before any of the other dialogues were displayed. This included who we (the authors) are, where the information came from and what our motives are. We also stated that we had no links to pharmaceutical companies or any other vested interests. The fact that both of our conditions included this information on trustworthiness, and that both of our conditions were similarly effective at increasing positive attitudes and intentions, implicitly suggests that being transparent about the source of information could be a crucial component for shifting vaccine attitudes and intentions. Of course, because our conditions did not differ in this way, this needs to be experimentally verified in future work. Nevertheless, the indirect comparison to Altay *et al*.'s results, in which the chatbot contained trust information and was more effective than the control that did not, further suggests communicating trust could be an important factor.

By design, the only difference between the current study's two conditions was that in the experimental condition participants had a choice over which information they saw, whereas in the control condition the information was shown at random. This suggests that having agency or ‘choice’ over the information one engages with may not be the most crucial aspect of why chatbots are effective. It suggests that addressing the concerns that are of most importance or interest to the participant may not be as crucial as previously thought, although it is important to note that all information was originally chosen to address common concerns of the vaccine-hesitant. Previous research suggests participants prefer choice and agency over information when given the choice, but perhaps this preference isn't enough to override the effectiveness of accurate and relevant information in general. Importantly, we did not find a difference in engagement ratings between our conditions, and participants spent a similar amount of time across both conditions. Again, when we compare to Altay *et al.*'s chatbot, we see that participants spent longer with their chatbot on average than with our information, and that time spent on the task is related to change in attitudes. These comparisons suggest that chatbots are most effective because of their ability to hold the attention of the participant and thus spend more time engaging with the information. Seemingly unimportant details of chatbots may account for their being engaging, than standard text, for example, the ‘social’ element of interacting with another ‘agent’ may be inherently more engaging, or simply the display of the information, which is often more ‘bitesize’ and delivered one sentence at a time.

It could be argued that our results are simply demonstrating a regression to the mean, particularly because we recruited from one end of the vaccination attitude spectrum, and we saw similar effects across both conditions. However, after investigating this possibility, it seems unlikely given that those who were rated as ‘against’ vaccination as opposed to ‘neutral’ were actually more likely to stay the same in their reported intention to get the vaccine than the neutral participants and were less likely than the neutral participants to increase their intention to get vaccinated (i.e. the opposite of what you would expect with regression to the mean). A plot displaying this is included in the electronic supplementary material. Furthermore, not only are our percentage changes very similar to Altay *et al*.'s chatbot condition effects, who were recruited from the general population and not specifically against vaccination, but also much greater than those in previous studies, for example when influenced by norms, participants only showed a 5% decline in rating themselves as ‘undecided’ or ‘against’ vaccination [4], whereas we found a 16% decline. Previous research also suggests that using question and answer (Q&A) style information is more effective than presenting pure fact-based information, again reporting similar effects to ours [3].

One aspect of our study worth noting is how the information was framed and how the participants were addressed throughout the study. Participants were asked if they were either against, for or neutral towards the COVID-19 vaccines, as it is worded in Prolific's pre-screening criteria. We thus used this as our wording and advertised the study as ‘Your opinions on COVID-19 vaccinations’. Part of our study (results not included for this publication) was to ask participants to imagine and put forward the opposite side's reasons for and against vaccination (this was conducted after their second round of attitude and intention measures, so would not influence the results of this study). We also offered participants an opportunity to provide any other feedback they had in an ‘anything else’ box. These comments were insightful, and often hinted that participants were keen to have an outlet for their views. Anonymity perhaps allowed them to be honest, and we also noted many thanked us for not referring to them, or anyone, as ‘anti-vaxxers’. We refrained from using this term throughout as this term is often used to stereotype or villainize those who hold those views. We wish to follow-up these comments, respond where necessary and share them with the rest of the research community to help further destigmatize those who are vaccine hesitant and help create an atmosphere of constructive dialogue and conversational receptiveness about these issues [17]. Comments are included in the Shiny App available at https://lottybrand.shinyapps.io/vaccineComments/.

Overall, we suggest it is important when designing science communication interventions to control for the amount of information, time spent with the information, trustworthiness of information and consequently to ensure a high level of engagement with the information. Simply providing Q&A style dialogues appeared to be as effective as delivering the same information via a chatbot, and more effective than previous studies using norms or simple fact-based interventions.

## Data Availability

All of our data, code and analysis scripts are available at https://github.com/lottybrand/clickbot_analysis. Data are also provided in the electronic supplementary material [18].
